# Structural analysis of missense mutations occurring in the DNA-binding domain of HSF4 associated with congenital cataracts

**DOI:** 10.1016/j.yjsbx.2019.100015

**Published:** 2019-11-15

**Authors:** Zaiyu Xiao, Ling Guo, Yang Zhang, Liwei Cui, Yujie Dai, Zhu Lan, Qinghua Zhang, Sheng Wang, Wei Liu

**Affiliations:** a College of Life Science and Technology, Huazhong University of Science and Technology, Wuhan, Hubei 430074, China; b Institute of Immunology, Army Military Medical University of PLA, Chongqing 400038, China; c Department of Obstetrics and Gynecology, Daping Hospital, Army Medical University of PLA, Chongqing 400038, China

**Keywords:** CC, congenital cataract, HSF, heat shock factor, DBD, DNA-binding domain, HSE, heat shock element, MD, molecular dynamics, Congenital cataract, Heat shock factor 4, DNA-binding domain, Molecular dynamics simulation

## Abstract

Congenital cataract (CC) is the major cause of childish blindness, and nearly 50% of CCs are hereditary disorders. HSF4, a member of the heat shock transcription factor family, acts as a key regulator of cell growth and differentiation during the development of sensory organs. Missense mutations in the HSF4-encoding gene have been reported to cause CC formation; in particular, those occurring within the DNA-binding domain (DBD) are usually autosomal dominant mutations. To address how the identified mutations lead to HSF4 malfunction by placing adverse impacts on protein structure and DNA-binding specificity and affinity, we determined two high-resolution structures of the wild-type DBD and the K23N mutant of human HSF4, built DNA-binding models, conducted *in silico* mutations and molecular dynamics simulations. Our analysis suggests four possible structural mechanisms underlining the missense mutations in HSF4-DBD and cataractogenesis: (i), disruption of HSE recognition; (ii), perturbation of protein-DNA interactions; (iii), alteration of protein folding; (iv), other impacts, e.g. inhibition of protein oligomerization.

## Highlights

• High-resolution structures of wild-type and K23N mutant DBD in HSF4 were determined.
• Cataract-related mutations in HSF4 were structurally analyzed through MD simulation.
• Mutations Q61R, K64E, R73H, R116H and R119C likely perturb DNA-binding activity.
• Mutations K23N, P60H and L114P probably affect trimer formation or folding dynamics.
• Mutations A19D, H35Y and I86V may be false positives leading to trivial impacts.

## Graphical abstract

**Figure:**
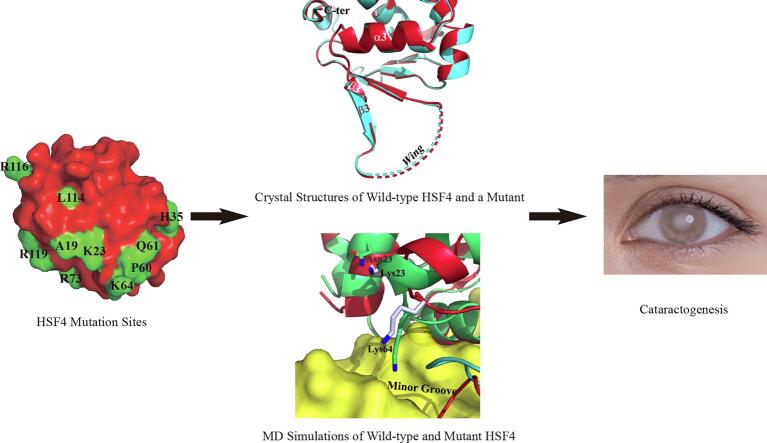

## 1 Introduction

Cataract is an eye disease arising from the loss of ocular lens transparency and can be classified as congenital and age-related according to the disorder onset. Congenital cataract (CCs) is a lens opacity present at birth and form the leading cause of childish blindness (Lund et al., 1992). Approximately 50% of CC cases may have a genetic background (Francis and Moore, 2004). It has been known that mutations in the genes encoding various crystallin proteins, cytoskeletal proteins, gap junction proteins or transcription factors, e.g. FOXE3, HSF4, MAF, and PITX3, may lead to CC formation (Anand et al., 2018, Hejtmancik, 2008, Shiels and Hejtmancik, 2017). According to recent sequencing and phenotyping data, the inheritance of CC may be familial and usually autosomal dominant (Anand et al., 2018, Berry et al., 2018).

HSF4 belongs to the heat shock factor (HSF) family conserved from fungi to humans. The members of this family serve as central regulators in maintaining cellular protein homeostasis or mediating cell differentiation and development (Akerfelt et al., 2010; D. Westerheide et al., 2012, Vihervaara and Sistonen, 2014). Malfunction of HSFs is often linked to severe human diseases such as cancer and neurodegenerative disorders (Dai et al., 2007, Neef et al., 2011, Scherz-Shouval et al., 2014). The human genome encodes six HSF proteins, among which HSF1, 2 and 4 have been extensively studied during past decades, but physiological functions of the others remain undetermined (Gomez-Pastor et al., 2018). HSF1 is the master regulator of the heat shock response against proteotoxic stress conditions, while HSF2 is more involved in specific developmental processes such as corticogenesis and spermatogenesis (Akerfelt et al., 2010, Vihervaara and Sistonen, 2014, Widlak and Vydra, 2017). HSF4 is not implicated in heat shock response but required for cell growth and differentiation during the development of sensory organs, e.g. eye lens, in cooperation with HSF1 (Fujimoto et al., 2008, 2004). There are two HSF4 isoforms, HSF4a and HSF4b, resulting from alternative splicing. HSF4b is the canonical isoform that contains an additional 30 amino acids and can activate transcription, while HSF4a inhibits the expression of other HSFs (Bjork and Sistonen, 2010, Pirkkala et al., 2001).

HSFs share similar module organizations in their sequences, which usually comprise an N-terminal DNA binding domain (DBD), an adjacent oligomerization domain also referred to as the hydrophobic repeat region (HR-A and -B), an intrinsically disordered regulatory domain and a C-terminal activation domain. DBD is the most conserved domain and by itself able to recognize an upstream promoter element, called the heat shock element (HSE), which is composed of multiple copies of inverted 5′-nGAAn-3′ (n can be any nucleotide) pentamers (Amin et al., 1988, Pelham, 1982, Xiao and Lis, 1988). The structure of DBD is characterized by a winged helix-turn-helix motif in its structural core (Feng et al., 2016b, Harrison et al., 1994, Vuister et al., 1994). When binding to a target gene, the second helix in this motif is inserted into the major groove of DNA and specifically contacts the GAA triplet present in an HSE (Jaeger et al., 2016, Littlefield and Nelson, 1999, Neudegger et al., 2016). Upon activation, HSF1 and HSF2 are converted into a DNA-binding component through homotrimerization or heterotrimerization. HSF4, however, is constitutively trimerized and thus has constant DNA-binding activity (Akerfelt et al., 2010, Gomez-Pastor et al., 2018). Each DBD in an HSF trimer binds to a single nGAAn repeat, and therefore three repeats are required for optimal binding (Gonsalves et al., 2011, Perisic et al., 1989, Xiao et al., 1991).

Despite being relatively less studied, HSF4 has arisen researchers’ extensive interest since a link of severe CC occurrence in Chinese and Danish families with 4 missense mutations in HSF4-DBD was reported (Bu et al., 2002). The correlation of this transcription factor with CC formation was further demonstrated in HSF4-deficient mice, which displayed inclusion-like structures in lens fiber cells soon after birth (Fujimoto et al., 2004, Min et al., 2004). After that, a number of mutations were identified in different ethnicities for both autosomal dominant and recessive congenital cataract (Table 1) (Anand et al., 2018, Berry et al., 2018, Enoki et al., 2010, Gillespie et al., 2014, Hejtmancik, 2008, Jing et al., 2014, Shi et al., 2008). Interestingly, all known autosomal dominant mutations lie within DBD, while recessive mutations are located outside (Anand et al., 2018, Berry et al., 2018). Despite the increasing number of identified CC-associated mutations, mechanistic understanding of how these mutations induce HSF4 malfunction is limited due to the insufficient biochemical/structural studies revealing clear pathways from genotypes to phenotypes.

**Table 1:** Published cataract-associated missense mutations in HSF4-DBD and their possible structural impacts.

Nucleotide Position	AA Position	Secondary Structure	Conservation	Cataract Class	Possible Structural Impact
c. 57C > A	A19D	Helix α1	High	Lamellar	NO
c. 69G > T	K23N	Helix α1	Very high	Cerulean	DNA-binding perturbation
c. 103C > T	H35Y	Loop	Very low	Congenital	NO
c. 179C > A	P60H	Helix α2	High	Nuclear	Protein misfolding
c. 182A > G	Q61R	Helix α2	Very low	Senile	Wing movement
c. 190A > G	K64E	Helix α2	Very high	Lamellar	DNA-binding perturbation
c. 218G > A	R73H	Helix α3	Very high	Congenital	HSE-recognition disruption
c. 256A > G	I86V	Wing	Very low	Senile	NO
c. 341 T > C	L114P	Helix α2	High	Lamellar	Protein misfolding
c. 355G > A	R116H	Helix α2	Very low	Lamellar	C-terminus movement
c. 362C > T	R119C	C-terminal loop	Very high	Lamellar	HSE-recognition disruption

*Notes*: AA = amino acid; NO = not obvious.

In this study, we attempted to explore conformational changes in HSF4-DBD and protein-DNA interactions induced by the CC-associated mutations. To this end, we determined the crystal structures of the wild type and a mutant DBD at atomic resolutions, built a DNA-binding model, conducted *in silico* mutations and molecular dynamics (MD) simulations. Our results suggest diverse mechanisms underlying the identified mutations in HSF4.

## 2 Materials and methods

### 2.1 Protein expression

The nucleotide sequence encoding the DNA-binding domain of human HSF4 (residues 17–122) were amplified by PCR and inserted into a pET-22b(+) plasmid (Novagen), which was subsequently transformed into *E. coli* host strain BL21(DE3). Bacteria were grown in LB medium containing 100 μg ml^−1^ ampicillin at 310 K. Expression of the recombinant protein was induced with 0.3 m*M* isopropyl β-D-1-thiogalactopyranoside (IPTG) for 4 h at 303 K.

### 2.2 Protein purification

Protein purification was conducted following a previously established protocol (Feng et al., 2016a). In short, the harvested bacteria were lysed using a high-pressure crusher at 277 K in the lysis buffer containing 50 mM NaH_2_PO_4_/Na_2_HPO_4_, pH 8.0, 500 mM NaCl and 25 mM imidazole. After removal of insoluble debris, the supernatant was loaded onto a HisTrap HP 5 ml column (GE Healthcare), which was eluted with 300 mM imidazole. The pooled fractions were then desalted and applied onto a HiTrap SP 5 ml column (GE Healthcare) pre-equilibrated with 20 mM HEPES pH 7.5, 100 mM NaCl. A linear-gradient elution with increasing NaCl concentration from 100 mM to 1.0 M was developed for protein elution. A step of size-exclusion chromatography was performed afterward to further improve the protein purification using a HiLoad 16/600 Superdex 75 column (GE Healthcare) and an elution buffer consisting of 20 mM Tris-HCl pH 8.0, 150 mM NaCl, 1 mM DTT, 0.2 mM EDTA and 5% glycerol.

The expression plasmid of the K23N mutant was built following the instructions of the Muta-direct™ Kit (SBS Genetech) using the pET22b-HSF4-DBD plasmid as the template. Subsequent expression and purification processes were same as those for the wild-type protein. The purified proteins were concentrated to 60 mg ml^−1^ and stored at 193 K until being used for crystallization.

### 2.3 Protein crystallization

The protein samples of wild-type DBD and the K23N mutant were diluted to 30 mg ml^−1^ before crystallization trials. Screening for initial crystallization conditions was carried out by means of sitting-drop vapor-diffusion using five commercial kits from Hampton Research (California, USA) and a Gryphon-LCP robot (Art Robbins Instruments, USA). Drops were set up by mixing 0.4 µl protein solution and 0.4 µl reservoir solution equilibrating with 40 µl reservoir solution in the well. The subsequent optimizations were manually conducted using the hanging-drop vapor diffusion method with drops consisting of 1 µl protein and 1 µl reservoir solution. Crystals of both the wild-type and mutant proteins were grown at 0.1 M Tris, pH 8.0 and 3.4 M sodium nitrate.

### 2.4 Diffraction data collection and processing

The crystals were directly mounted in nylon cryoloops (Hampton Research) and flash-cooled in a stream of liquid nitrogen. X-ray diffraction data for the wild-type DBD and the K23N mutant were collected on beamline BL19U1 and BL18U1, respectively, at Shanghai Synchrotron Radiation Facility (SSRF) in China. A total of 360 images with 1° oscillation step per frame were recorded. All diffraction data were processed using *HKL*-3000 (Minor et al., 2006).

### 2.5 Structure determination and refinement

The structure of wild-type HSF4-DBD was determined by means of molecular replacement using the recently solved structure of human HSF1-DBD (PDB entry of 5HDG) (Feng et al., 2016b) as a search model. After the automatic model building using *Phenix.autobuild* (Terwilliger et al., 2008), the structure was refined using *Phenix.refine* (Afonine et al., 2012) with several rounds of manual remodeling between refinement cycles using the modeling toolkit *Coot* (Emsley et al., 2010). Structure of the K23N mutant was solved by molecular replacement as well using the wild-type structure as a search model, and refined in the same way. Statistics of data collection and structure refinement are summarized in Table 2. All structural representations were generated using the molecular visualization program *PyMOL* (Schrodinger LLC, 2015).

**Table 2:** Data collection and refinement statistics.

	HSF4-DBD	K23N mutant
Data collection		
Space group	*I*222	*P*22_1_2_1_
Cell parameters		
*a*, *b*, *c* (Å)	44.62, 68.23, 80.10	44.63, 65.05, 80.56
α, β, γ (°)	90, 90, 90	90, 90, 90
Resolution (Å)	38.98–1.20 (1.22–1.20)	27.17–1.69 (1.73–1.69)
*R*_sym_	0.049 (0.452)	0.070 (0.668)
*I*/σ(*I*)	49.3 (3.5)	24.1 (3.9)
Completeness (%)	99.3 (89.1)	99.4 (99.8)
Redundancy	12.3 (6.7)	6.8 (6.8)
Wilson plot B	11.3	8.9
Refinement		
No. reflections	36,700	26,197
*R*_work_/*R*_free_	0.133/0.151	0.171/0.205
No. atoms		
Protein	780	1574
Ions	14 (Na^+^, NO_3_^–^)	16 (Na^+^, NO_3_^–^)
Water	127	240
*B*-factors		
Protein	20.55	22.38
Ions	20.02	15.39
Water	41.11	34.47
R.m.s. deviations		
Bond lengths (Å)	0.007	0.012
Bond angles (°)	0.903	1.207
Ramachandran plot		
Favored (%)	100	99.44
Allowed (%)	0	0.56
Outliers (%)	0	0
**PDB ID**	6J6V	6J6W

*Values in parentheses are for the highest resolution shell.

### 2.6 Modeling of HSF4-DBD bound to DNA and *in silico* mutation

To generate a model of HSF4-DBD bound to DNA similar to the recently reported structures of HSF1/2-DBDs in complex with DNA containing two binding sites (Jaeger et al., 2016, Neudegger et al., 2016), PDB entry 5D8K was used as the template. The two HSF2-DBDs existing in that structure were replaced with the refined structure of HSF4-DBD through superimposition, and thus a model of two DBD copies bound to a palindromic 12-bp DNA duplex containing TTCtaGAA at the center was generated. After auto-building of the missed wing using homology modeling, energy minimization, and short-time MD simulation were performed to exclude steric conflicts. The DNA molecule in this model was then replaced by a 24-bp B-form ds-DNA comprising the above palindromic sequence at the middle region generated using *Nucgen* from the *Amber*14 package (Case et al., 2014). One DBD copy in the resultant model was subjected to *in silico* mutations including A19D, K23N, H35Y, L114P, Q61R, K64E, I86V, and R116H, while the other remained unchanged. The model remaining wild type in both DBDs served as the reference system for MD simulations.

### 2.7 MD simulations

Simulations were performed using the *Amber*14 package (Case et al., 2014) in parallel on an 8-GTX1080Ti-GPU workstation following a five-step protocol including ensemble construction, minimization, heating, equilibration, and production. The force field parameters retrieved from ff14SB and ff99bsc0 for protein and DNA respectively were applied. Each ensemble was explicitly solvated in a box using the TIP3 water model. An appropriate amount of Na^+^ and Cl^-^ ions were added to each ensemble to mimic the physiological saline milieu and ensure zero net charges. After minimization, heating, and equilibration, one 100 million steps of NPT simulations with 1-fs step size at 300 K were calculated in the process of production. The embedded tools in *Amber* were used to analyze the resultant trajectories from which a final stable conformation for each DBD-DNA complex was extracted. For structural comparison, all mutant models were aligned to the output coordinates from the reference system.

## 3 Results

### 3.1 The DBD in HSF4 displays a conserved overall structure with other HSFs

**Fig. 1:**
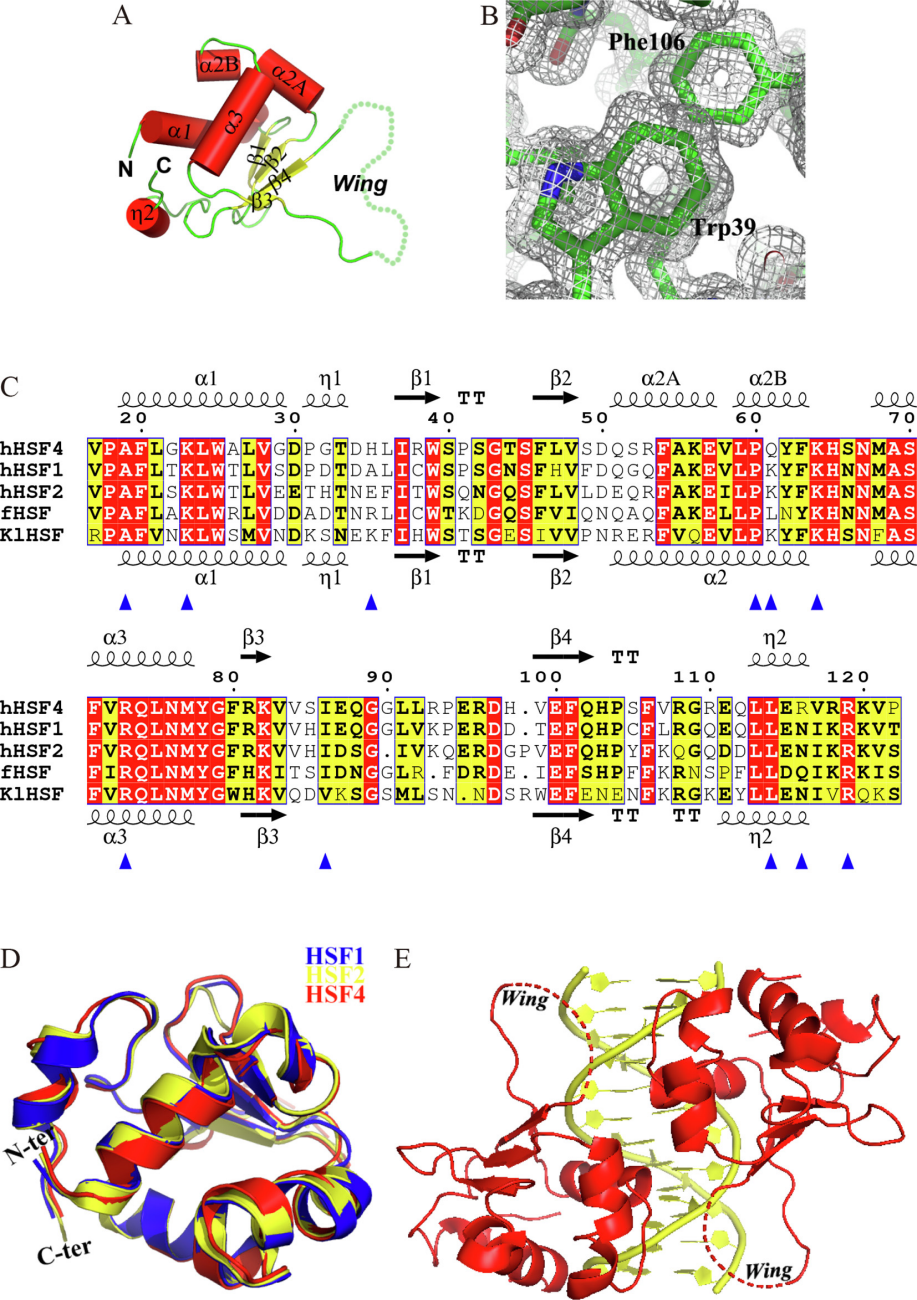
The overall structure and amino acid sequence of the DNA-binding domain in HSF4. (A) The structural topology of HSF4-DBD. Helixes and β-strands are represented by red cylinders and yellow arrows, respectively. (B) Representative 2*F*_o_ – *F*_c_ density contoured at 1.0 σ. (C), Sequence comparison among structurally characterized DBDs in human HSF4, HSF1, HSF2, fruit fly HSF and *Kluyveromyces lactis* HSF. Secondary structures of HSF4 and HSF1 are schematically shown at the top and bottom of the aligned sequences, respectively. Reported CC-associated mutation sites occurring in HSF4-DBD are labeled by blue triangles. (D), Structural superimposition of human HSF1, HSF2, and HSF4. (E), A DNA-binding model containing two HSF4-DBD copies. (For interpretation of the references to color in this figure legend, the reader is referred to the web version of this article.)

HSF4-DBD (residues 17–122) was crystallized in space group *I*222, same as the DNA-free structure of HSF1-DBD determined recently in our lab (PDB entry 5HDG) (Feng et al., 2016b). The diffraction resolution at 1.2 Å, which was the highest among all available HSF structures, allowed us to refine a model with excellent quality (Table 2). The asymmetric unit contains a single monomer exhibiting a characteristic fold comprising the winged helix-turn-helix motif (Fig. 1A). Under such a resolution, the 2*F*_o_ - *F*_c_ density was so sharp to define the precise position of each non-hydrogen atom (Fig. 1B), although residues within the wing (residues 88–96) were completely invisible and had to be omitted from the model (Fig. 1A), which is consistent with most reported DBD structures (Feng et al., 2016b, Harrison et al., 1994, Littlefield and Nelson, 1999, Neudegger et al., 2016).

HSF4-DBD shares high degree of sequence homology and identical structural topology with other HSF-DBDs, in particular with human HSF1 (sequence identity = 77%, RMSD = 0.697 Å) and HSF2 (sequence identity = 70%, RMSD = 0.617 Å) (Fig. 1C and 1D). Only slight structural differences were observed, e.g. discontinued helix α2 in HSF4 vs integrated one in other HSFs. For further analysis of the CC-associated mutations, a DNA-binding model was built by replacing HSF2-DBD in the PDB entry of 5D8K (Jaeger et al., 2016) with the refined model of HSF4-DBD. In this model, two copies of HSF4-DBD bind to a palindromic DNA molecule containing two binding sites arranged in the tail-to-tail orientation (Fig. 1E). Starting from this model, we conducted *in silico* mutations in only one DBD copy followed by MD simulations for 100 ns, with all ensembles displaying reasonable RMSD fluctuations (Supplementary Figs. 3–5).

### 3.2 CC-associated mutations occur in either highly or poorly conserved positions

**Fig. 2:**
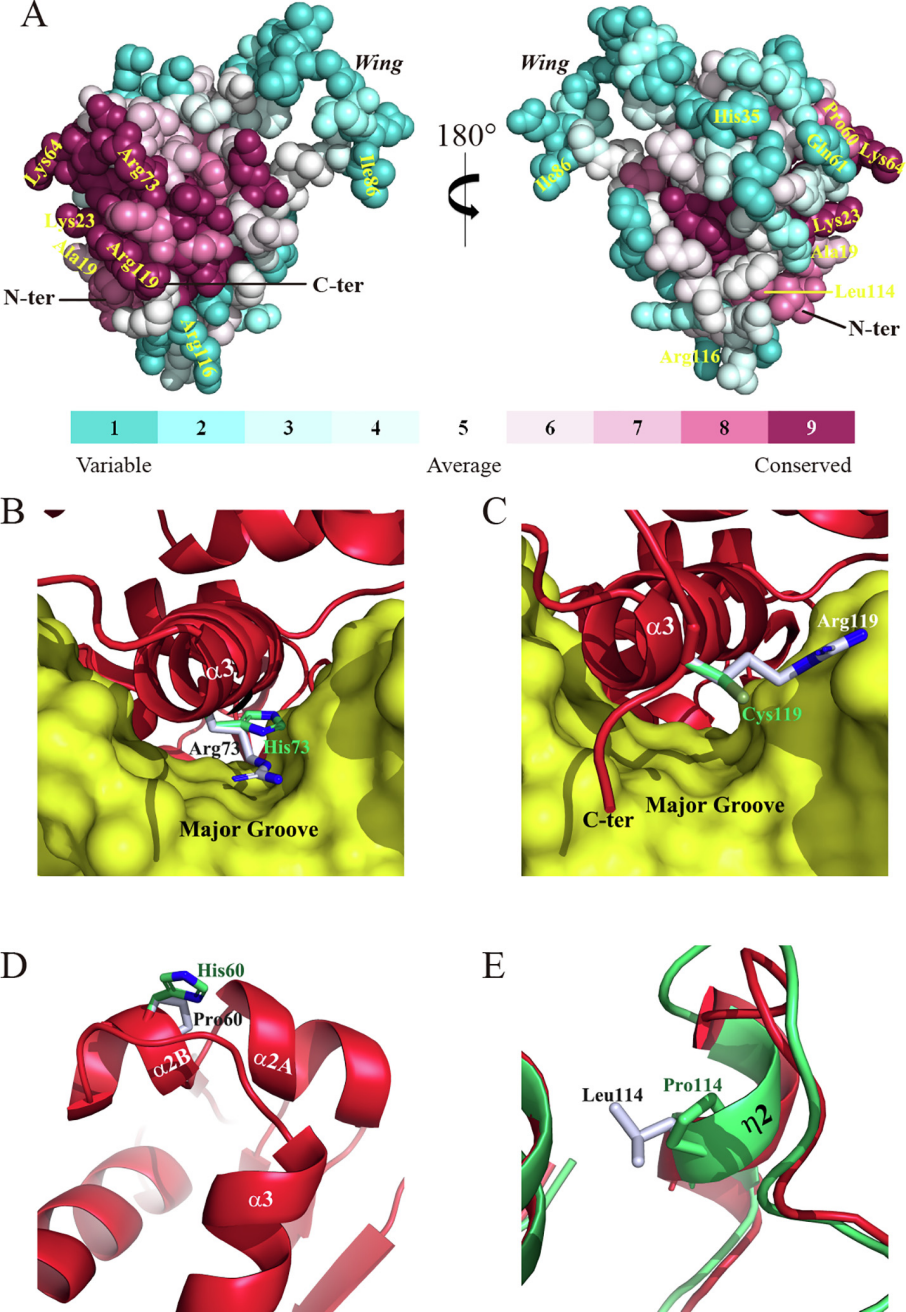
Relative conservation of individual amino acids and modeling of the R73H, R119C, P60H, and L114P mutants. (A) The conservation map of HSF4-DBD calculated using the *ConSurf* server (Landau et al., 2018). Protein atoms are displayed as spheres with color-indexes according to conservation scores and CC-associated mutations labeled. (B–D) Modeling of the R73H (B), R119C (C) and P60H (D) mutants without simulation. (E) The model of the L114P mutant after simulation. Side chains are shown as sticks with carbon atoms colored in bluewhite for wild-type residues and green for mutated residues. Nitrogen and sulfur atoms are colored in blue and flavogreen, respectively. The cartoon models representing the wild-type and mutant DBDs are colored in red and green, respectively. The accessible surface of DNA molecules is colored in yellow. (For interpretation of the references to color in this figure legend, the reader is referred to the web version of this article.)

To analyze relative conservation of the mutation site associated with congenital cataract, the sequence and structure of HSF4-DBD was input to the *ConSurf* server (Landau et al., 2018), where a conservation profile of individual amino acids was generated by psi-blasting with 150 homologous sequences retrieved from the REF90 database (Suzek et al., 2007). The reported positions can be classified into three groups (Table 1 and Supplementary Fig. 1): I, invariant (K23, K64, R73, and R119); II, highly conserved (A19, P60 and L114); III, non-conserved (H35, Q61, I86 and R116), meaning that no CC-associated mutations occur at moderately conserved positions. As expected, most conserved amino acids (group I and II) are exposed on the surface assumingly contacting DNA, while all poorly conserved residues are present on the DNA-distal surface (Fig. 2A). The only exception is L114, a partially buried hydrophobic residue.

### 3.3 Mutations R73H and R119C very likely destroy HSE recognition

R73 and R119 are highly conserved Arg residues presumably contacting bases within the major groove (Jaeger et al., 2016, Neudegger et al., 2016). Their counterparts in human HSF2, R63, and R109 for example, respectively form bidentate hydrogen bonds with the guanine in GAA (Supplementary Fig. 2A) and a solvent-mediated hydrogen bond with the purine preceding the TTA triplet in the complementary strand (Supplementary Fig. 2B). The guanidine side chains of these two arginines are necessary for the conserved hydrogen bonds essential for HSE recognition. We hence reason that any replacement of either R73 (Fig. 2B) or R119 (Fig. 2C) would very likely abolish the activity to recognize HSEs by destroying the base-specific interactions.

### 3.4 Mutations P60H and L114P probably affect protein folding

As an imide acid, proline is rarely found at internal positions in helices, which would otherwise mediate the formation of kinks because its cyclic pyrrolidine side-chain restricts the dihedral angles of the preceding amino acid (Barlow and Thornton, 1988). A strictly conserved Pro residue, however, is present at the center of a bulged kink in helix α2 in HSFs (Feng et al., 2016b, Harrison et al., 1994, Vuister et al., 1994), e.g. P60 in HSF4 (Fig. 2D), and was found to be irreplaceable in maintaining proper folding and protein solubility (Hardy and Nelson, 2000). Based on that study, it seems likely that the mutation of P60H may lead to insoluble HSF4 expression due to aberrant folding kinetics.

L114 is a buried hydrophobic amino acid located in a 3_10_ helix (η2) close to the C-terminus of HSF4-DBD. The occurrence of the L114P mutation means that a Pro residue is introduced at the internal of a 3_10_ helix, which would generally destruct the helix integrity because of the restricted torsion angles. Consistently, MD simulation showed the deformation of η2 from a two-turned to a single-turned helix upon this mutation (Fig. 2E). Considering the role of P60 in protein folding, we suppose that the replacement of L114 with a Pro residue may also alter the solubility of newly synthesized HSF4.

### 3.5 Crystal structure of the K23N mutant reveals significant conformational changes

**Fig. 3:**
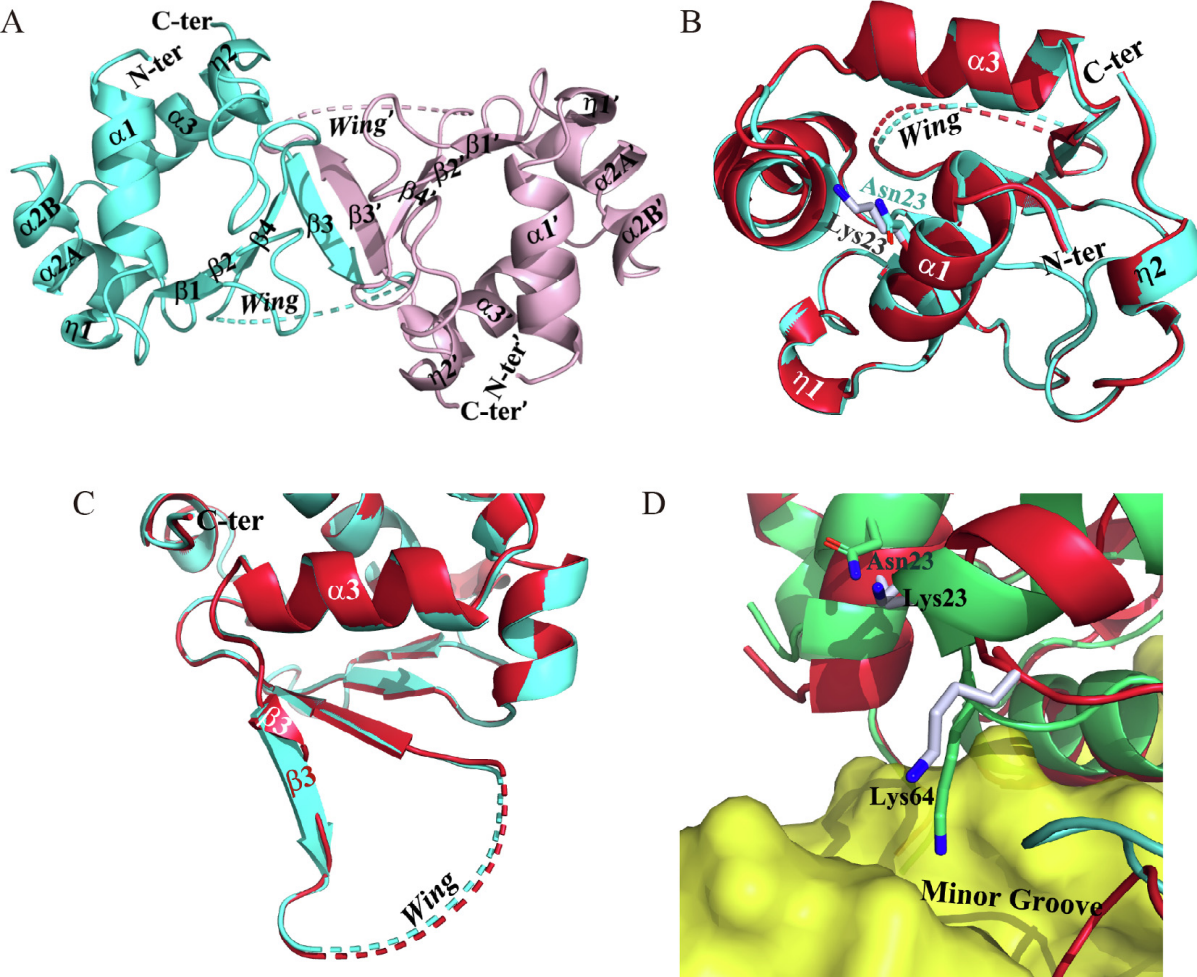
Crystal structure and MD simulation of the K23N mutant. (A) Two monomers sitting in the asymmetric unit. (B and C) Superimposition of the wild type DBD (red) and the K23N mutant (cyan) showing overall similarity (B) and a local difference in stand β3 (C). (D) MD simulation of the wild type DBD (red) and the K23N mutant (green) bound to DNA (yellow). Carbon atoms in side chains are colored in bluewhite for the wild type and green for the mutant, respectively. (For interpretation of the references to color in this figure legend, the reader is referred to the web version of this article.)

K23 is a highly conserved amino acid located in helix α1, but spatially close to the C-terminus of helix α2. We crystalized this mutant in space group *P*22_1_2_1_ (Table 2). The asymmetric unit accommodates two protein monomers with an extended hybrid β-sheet formed at the dimeric interface (Fig. 3A). The overall scaffold of the mutant showed high similarity with the wild-type protein (RMSD = 0.186 Å) (Fig. 3B). A striking difference, however, was observed in stand β3, which became much longer than that in the wild-type DBD (Fig. 3C). The change occurring in this β-strand gives more constraints to the downstream wing loop, which was, though, invisible in density.

We further built a DNA-binding model using the refined structure and did an MD simulation. Compared with the wild-type protein, helix α2 moves along with the helical axis to its C-terminus, and the side chain of K64 is inserted into the DNA minor groove, deviating from the horizontal orientation over the minor groove in the wild-type DBD (Fig. 3D). This comparison means that upon the K23N mutation, conserved interactions of K64 with the DNA backbone are replaced by contacts with bases, which is probably adverse to HSE recognition. In another regard, considering that the wing is involved in protein-protein interactions in an HSF trimer (Jaeger et al., 2016, Littlefield and Nelson, 1999, Neudegger et al., 2016), we speculate that this mutant with a more constrained wing may exhibit weaker DNA-binding affinity due to deterioration of the synergetic binding to multiple repeats within an HSE.

### 3.6 Mutations Q61R, K64E, and R116H may perturb DNA-binding by inducing conformational changes in local regions

**Fig. 4:**
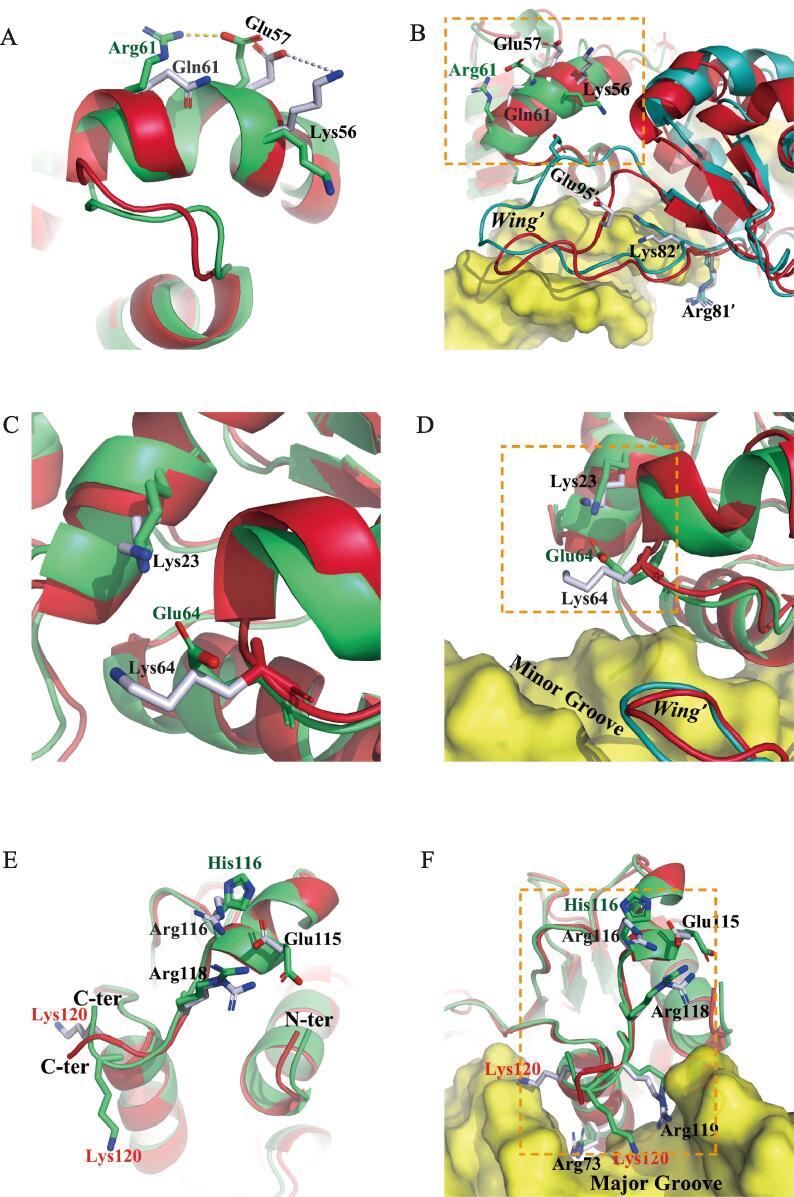
MD simulations of the Q61R, K64E and R116H mutants. In all panels, cartoons of the wild-type and mutant DBD are colored in red and green, respectively; the molecular surface of the DNA duplexes are colored in yellow. (A - C) Dimeric superimposition of the wild type DBD and the Q61R (A), K64E (B) and R116H (C) mutant bound to DNA (right) after simulation, and closer views of the dashed box therein (left). The neighboring DBD remaining in wild type is colored in cyan. (For interpretation of the references to color in this figure legend, the reader is referred to the web version of this article.)

Q61 is a poorly conserved amino acid in helix α2 (Fig. 1C), the first helix in the helix-turn-helix motif. When Q61 is replaced by arginine, cascaded conformational changes happened during a simulation. A new slat bridge between R61 and E57 replaced an old one between two preceding residues, K56 and E57, and the side chain of K56 was pushed away (Fig. 4A). Subsequently, E95′ in the neighboring DBD was pulled closer to reoriented K56, which further led to an apparent wing movement in that DBD, and as a result, the side chain of K82′ was inserted into the DNA major groove (Fig. 4B). The net consequences of these changes were varnished contacts between K82′ and the DNA backbone, perturbation of base-specific contacts in the major groove, and probably devastated wing-mediated protein-protein interactions.

K64 is a highly conserved amino acid in the turn connecting helices α2 and α3, which is involved in electrostatic contacts with the DNA phosphates (Jaeger et al., 2016, Neudegger et al., 2016). K64E is a pronounced replacement from a positive charge to a negative charge. According to our simulation for this mutant, a salt bridge was formed between E64 and K23 in the vicinity, which led to a slight movement of helix α2 (Fig. 4C). More strikingly, the HSF4-DNA affinity greatly drops due to the repulsive force between the negative charges in E64 and the DNA backbone (Fig. 4D).

The C-terminal peptide of HSF4-DBD (residues 114–122) presumably wraps the DNA duplex and directs the orientation of the coiled-coil formed in an HSF4 trimer (Jaeger et al., 2016, Neudegger et al., 2016). This peptide comprises an arginine/lysine cluster that contributes to either a base-specific contact in the major groove (e.g. R119) or electrostatic contacts with the DNA phosphates (e.g. R118 and K120). Its conformation is stabilized by charge-charge interactions among E115, R116, and R118, which is however destabilized by the mutation of R116H. As revealed in the MD simulation, rotation of the E115 side chain by almost 180**°** induced local conformational change propagated to the very end of DBD, which let the side chain of K120 deviated from the DNA backbone (Fig. 4E and 4F). As a result, the crucial contacts between the C-terminal peptide and DNA were significantly impaired upon this mutation.

### 3.7 Mutations A19D, H35Y, and I86V induces unnoticeable conformational changes and insignificant impacts on DNA-binding

**Fig. 5:**
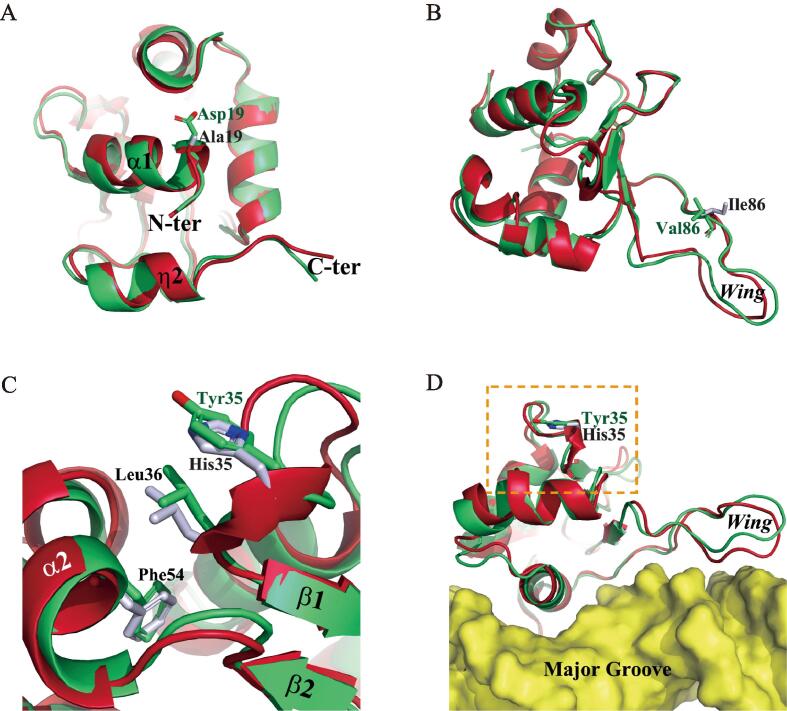
MD simulations of the H35Y, I86V and A19D mutants. In all panels, the backbone of the wild-type and mutant DBD are colored in red and green, respectively, while the molecular surface of the DNA duplexes is shown in yellow. (A and B) Superimposition of the wild-type DBD and the A19D (A) and I86V (B) mutants after simulation. (C and D) Superimposition of the wild-type DBD and the H35Y mutant after simulation, displaying local (C) and overall (D) conformational differences. (For interpretation of the references to color in this figure legend, the reader is referred to the web version of this article.)

As a conserved amino acid at the N-terminus of helix α1, A19 is not involved in protein-DNA interactions (Jaeger et al., 2016, Littlefield and Nelson, 1999, Neudegger et al., 2016). Mutation to an aspartic acid introduces a negative charge at this position, which however unexpectedly induces minute changes in either local or overall conformation, according to the simulation performed for this mutant (Fig. 5A).

H35 and I86 are poorly conserved amino acids exposed on the surface distal to DNA (Fig. 2A). According to the simulations, the replacement of I86 with a valine residue did not change the hydrophobic property at this position, which merely induced trivial changes in the wing (Fig. 5B). Slightly differently, the mutation of H35 to a tyrosine prompted the formation of a local hydrophobic core with the involvement of L36 and F54 (Fig. 5C). The overall conformation and protein-DNA interactions, however, seemed barely affected by this mutation (Fig. 5D).

## 4 Discussion

A number of mutations in human HSF4-encoding gene have been reported linking with cataractogenesis (Table 1) (Anand et al., 2018, Berry et al., 2018, Bu et al., 2002, Francis and Moore, 2004, Jing et al., 2014, Shi et al., 2008, Shiels and Hejtmancik, 2017). Protein trimerization, DNA-binding and transcriptional activities of some HSF4 mutants have been probed using chemical cross-linking, electrophoretic mobility shift assay (EMSA) and reporter gene transcription (Enoki et al., 2010), but detailed mechanisms underlining the pathways from genotypes to phenotypes remain to be determined. To address how the reported mutations impair HSF4 functions, we determined high-resolution structures of the wild-type DBD and the K23N mutant, built a dimeric DNA-binding model, conducted *in silico* mutations and carried out molecular dynamics simulations (Supplementary Figs. 3–5). Our analysis indicates that the mutations lead to activity loss probably through diverse mechanisms, which can well account for previous experimental observations (Enoki et al., 2010).

R73 and R119 are two irreplaceable arginine residues playing key roles in HSE recognition and binding. The mutations on them, R73H and R119C, destroy the base-specific interactions occurring in the major groove of DNA (Fig. 2B and 2C), and thus definitely inhibit DNA-binding and transcription activity, which is fully convergent with the observations from Enoki et al. (2010).

The two mutations with proline involvement, however, seem more likely to cause improper folding of HSF4 (Fig. 2D and 2E). The P60H mutation was identified very recently (Li et al., 2016). Although the experimental data for this mutant are unavailable, we believe that this mutation likely results in protein insolubility according to a previous mutational study of yeast HSF (Hardy and Nelson, 2000). Despite being expressed in soluble form, the L114P mutant showed severely inhibited trimerization, DNA-binding and reporter gene transcription (Enoki et al., 2010), and could not be crystallized (data from this work), all inferring significant differences with the wild-type DBD in terms of protein structure and stability, which are usually associated with protein folding.

The MD simulations of the K23N, Q61R, K64E, and R116H mutants suggest that these mutations probably impair DNA-binding specificity and/or affinity by inducing conformational changes in local regions (Fig. 3, Fig. 4). The activity loss of the K23N and K64E mutants has not been reported yet, but their structural impacts are straightforward, both of which abolish the charge-charge interactions between K64 and DNA phosphates (Fig. 3D and 4D). More interestingly, the crystal structure of K23N reveals a marked extension of stand β3, which in principle renders the wing loop less flexible (Fig. 3C). Although this conformation deviation from the wild-type protein might reflect a crystal-packing artifact, there is a possibility that this mutation may also damage the synergic DNA binding among DBDs in an HSF4 trimer, which is usually mediated by the wing (Jaeger et al., 2016, Littlefield and Nelson, 1999, Neudegger et al., 2016). In agreement, relatively weakened DNA-binding activity of this mutant compared with the wild-type protein was detected by EMSAs (Supplement Fig. 6). Mutations Q61R and R116H were originally found in age-related cataracts but later confirmed to cause childhood lamellar cataract in transgenic mice (Jing et al., 2014). These mutations occurring at non-conserved positions (Fig. 2A) did not show notable differences with the wild type protein in trimerization, DNA binding, and transcription activity (Enoki et al., 2010). The simulations performed in this work, however, revealed perturbations of interactions between neighboring charged residues and the DNA backbone (Fig. 4B and 4F). Perhaps such perturbations are not so intense to be observed in EMSAs but may affect the transcription of some genes containing non-canonical HSEs in the promoter.

Dissimilarly, mutations A19D, H35Y, and I86V seem to induce insignificant conformational changes or impacts to DNA-binding (Fig. 5). The simulations for H35Y and I86V, which occur at non-conserved positions on the DNA-distal surface (Fig. 2A), were in good consistency with the EMSA and transcription assays displaying unnoticeable difference from wild-type HSF4 (Enoki et al., 2010). Considering that these two mutations were detected together with others (Bu et al., 2002, Gillespie et al., 2014), perhaps either of them alone is not a sufficient cause for cataract. The A19D mutation, however, obviously inhibits HSF4 trimerization and the transcription of the *GRYGC* gene encoding γC-crystallin (Enoki et al., 2010). A19 is a conserved amino acid in helix α1, which possibly acts as a trimerization regulator (Tateishi et al., 2009). We thus speculate that the replacement of A19 with an aspartic acid may prevent trimer formation due to the repulsive force between the negative charges introduced in helix α1 in different HSF4 subunits.

In summary, the CC-associated mutations occurring in the DNA-binding domain of HSF4 may lead to cataract formation through diverse mechanisms including (i), disruption of HSE recognition (R73H or R119C); (ii), perturbation of protein-DNA interactions (K23N, Q61R, K63E, or R116H); (iii), alteration of protein folding (P60H or L114P); (iv), other probable impacts, e.g. inhibition of protein oligomerization (A19D). The mutation of H35Y or I86V, however, may not be a sufficient cause alone for cataractogenesis. In the context of a general lack of mechanistic studies between mutation identification and disease occurrence, our structural analysis performed for HSF4 may serve as a methodological study in future researches bridging genotyping and phenotyping.

## Declaration of Competing Interest

The authors declare that they have no known competing financial interests or personal relationships that could have appeared to influence the work reported in this paper.
