# Supplementary material for: Structural analysis of missense mutations occurring in the DNA-binding domain of HSF4 associated with congenital cataracts
Source: J Struct Biol X. 2019 Nov 15;4:100015. doi: 10.1016/j.yjsbx.2019.100015 (PMC7337047; doi:10.1016/j.yjsbx.2019.100015)
Supplement: Supplementary data 1 [file mmc1.docx]

**Structural analysis of missense mutations occurring in the DNA-binding domain of HSF4 associated with congenital cataracts**

Zaiyu Xiao^1§^, Ling Guo^2§^, Yang Zhang^1^, Liwei Cui^2^, Yujie Dai^3^, Zhu Lan^2^, Qinghua Zhang^3*^, Sheng Wang^1*^ and Wei Liu^2*^

^1^ College of Life Science and Technology, Huazhong University of Science and Technology, Wuhan, Hubei, 430074, China

^2^ Institute of Immunology, Army Military Medical University of PLA, Chongqing, 400038, China

^3^ Department of Obstetrics and Gynecology, Daping Hospital, Army Medical University of PLA, Chongqing 400038, China.

^§^ These authors contributed equally to this work.

^*^ Correspondence: [zhangqh1123@163.com](mailto:zhangqh1123@163.com) (Q. Zhang), [shengwang@hust.edu.cn](mailto:shengwang@hust.edu.cn) (S. Wang) and [wei.liu.2005@gmail.com](mailto:wei.liu.2005@gmail.com) (W. Liu).

**Corresponding Author: Zaiyu Xiao**

College of Life Science and Technology, Huazhong University of Science and Technology, Wuhan, Hubei, 430074, China

Email: [853695019@qq.com](mailto:853695019@qq.com)

Telephone: 18883244236


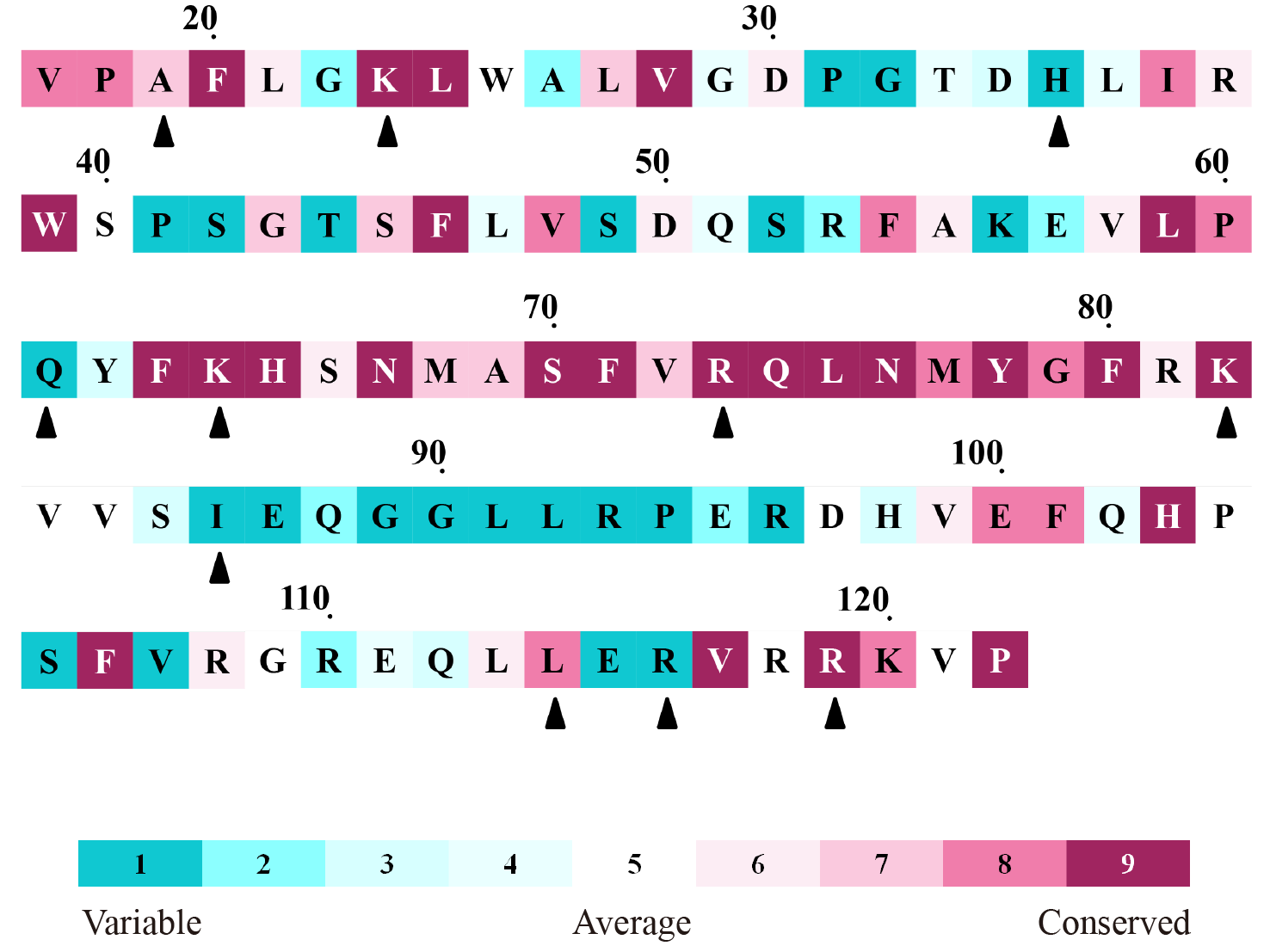


**Supplementary Figure 1.** Relative conservation of individual amino acids in the sequence of HSF4-DBD. Amino acids are colored according to the conservation scores calculated from the ConSurf server. Reported cataract-related mutation sites occurring in HSF4-DBD are highlighted by beneath black triangles.


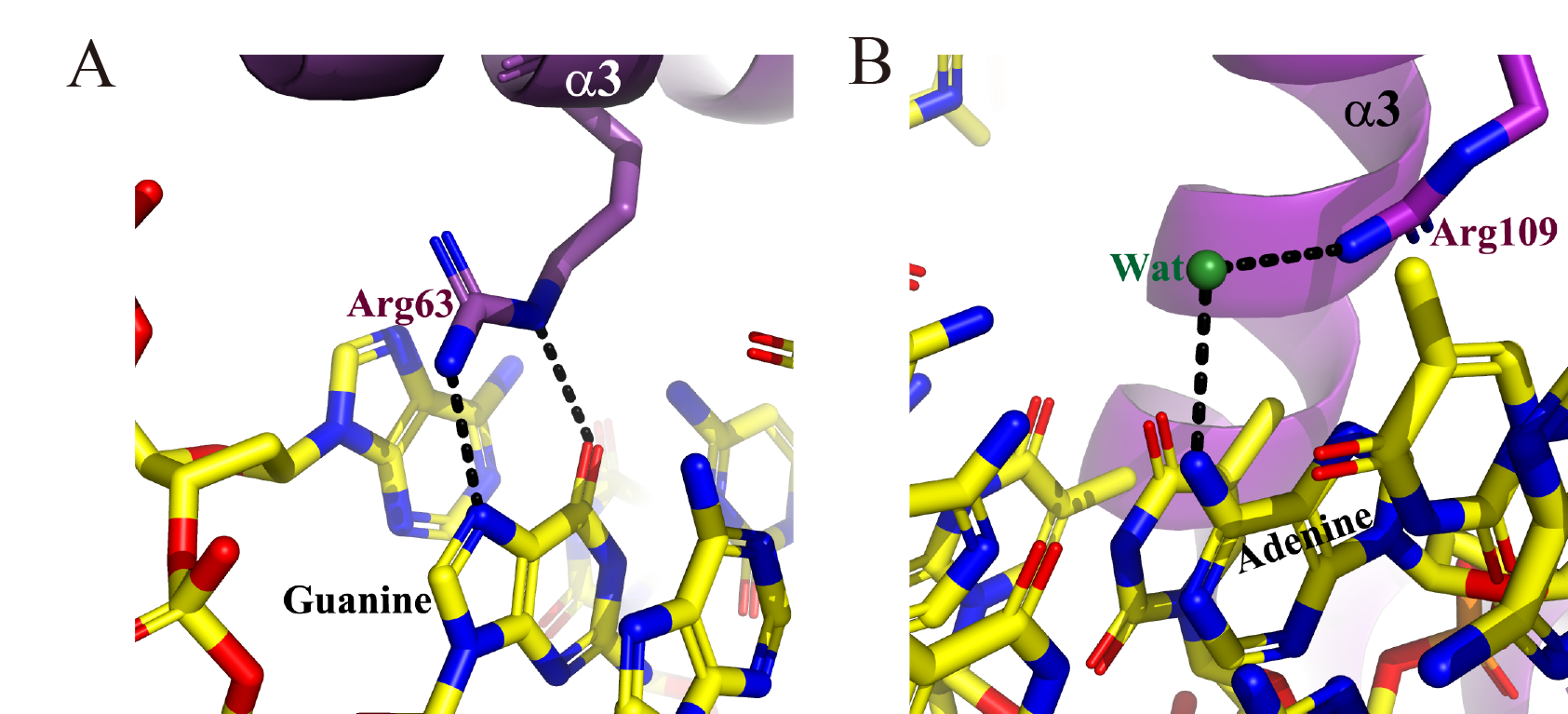


**Supplementary Figure 2.** Base-specific interactions present in the major groove of DNA bound by HSF2-DBD (PDB entries 5d8k (A) and 5d8l (B)). (A) Bidentate hydrogen bonds formed between Arg63 and the guanine of GAA in an HSE. (B) Solvent-mediated hydrogen bonds formed between Arg109 and a purine (usually adenine) upstream to the TTC triplet in the complementary DNA strand. In both panels, hydrogen bonds are represented as dash lines.


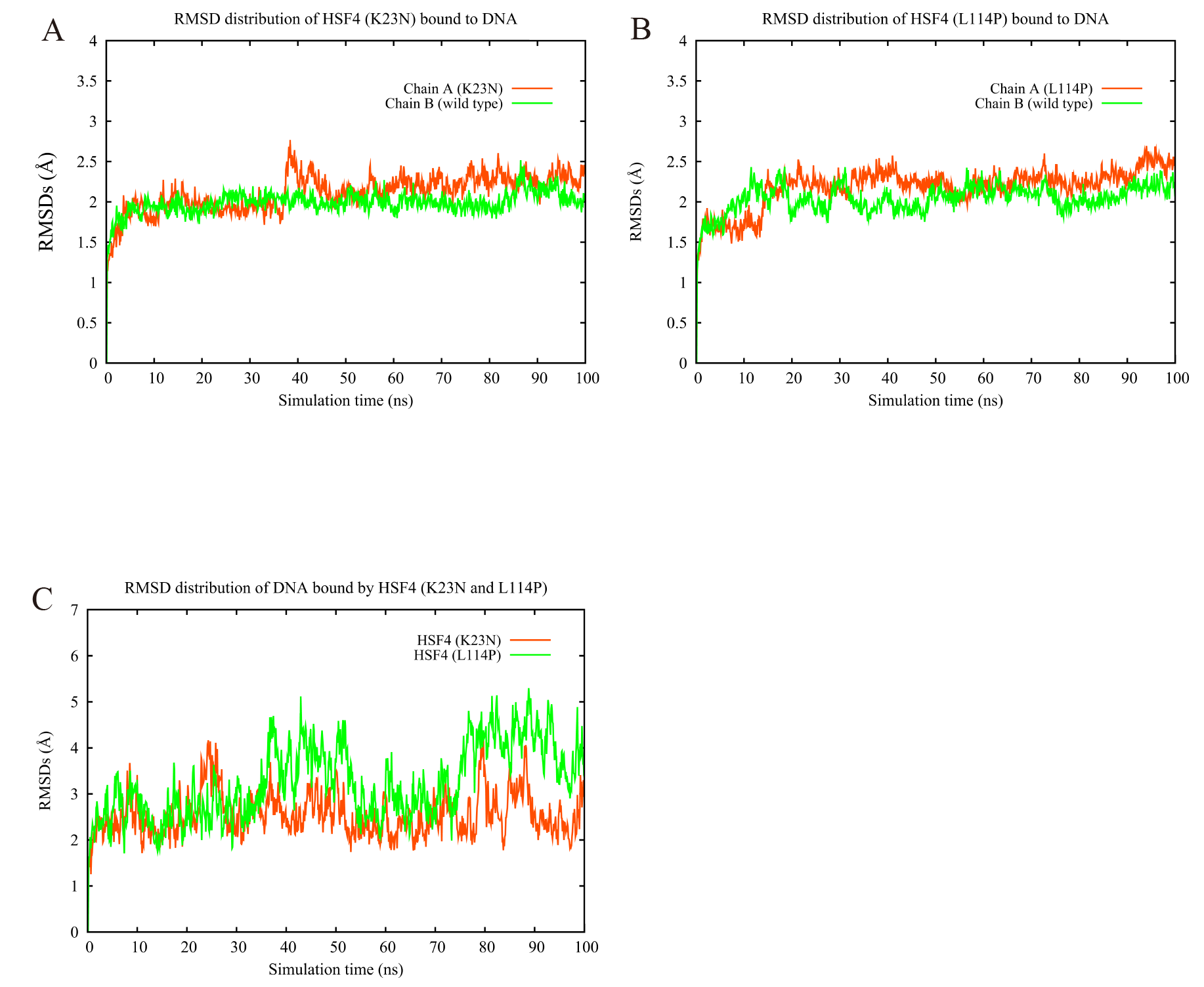


**Supplementary Figure 3.** RMSD distributions during MD simulations of the DBD carrying the K23N (A) and L114P (B) mutations, and the DNA molecules in the corresponding complex models (C).


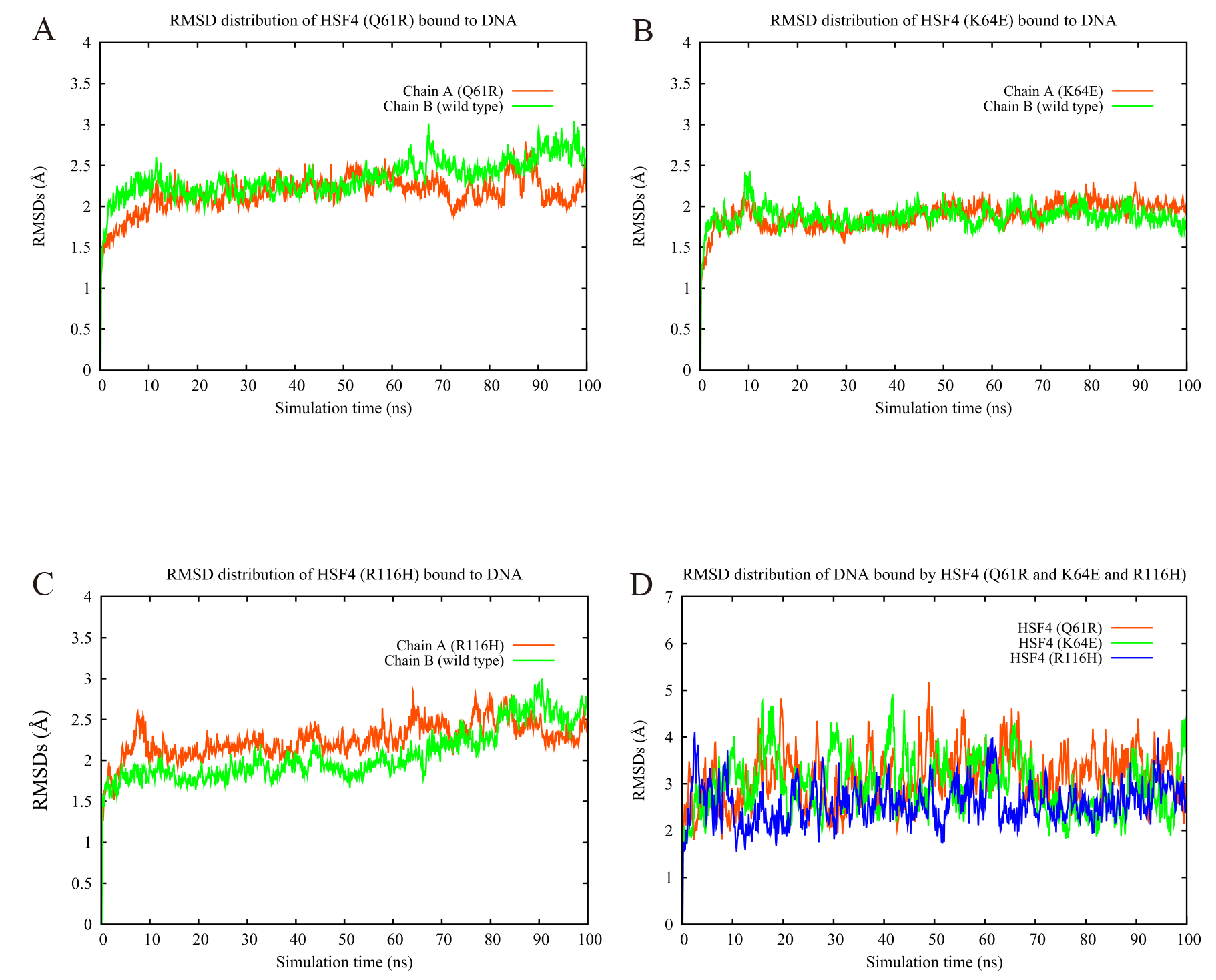


**Supplementary Figure 4.** RMSD distributions during MD simulations of the DBD carrying the Q61R (A), K64E (B) and R116H (C) mutations and the DNA molecules in the corresponding complex models (D).


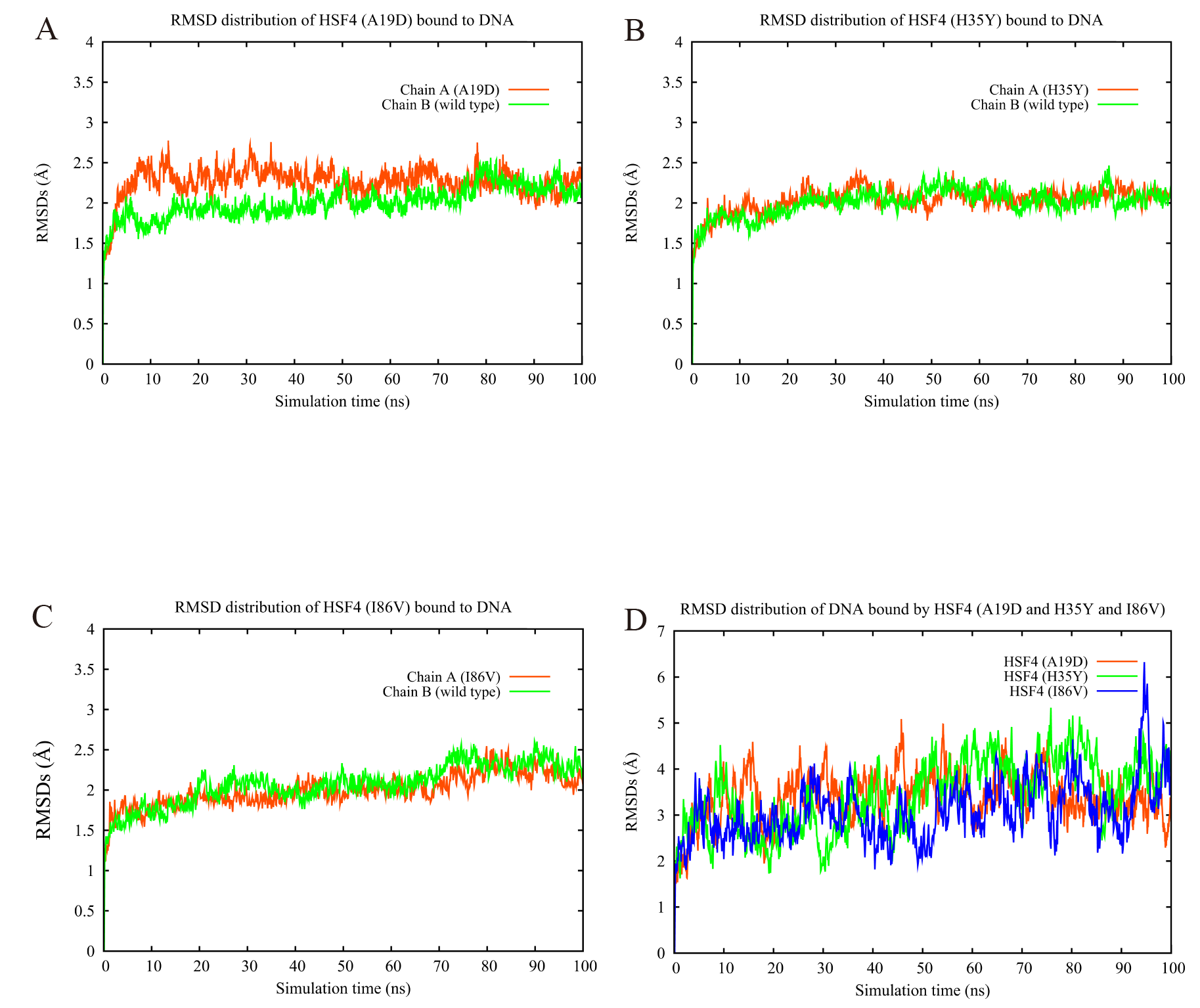


**Supplementary Figure 5.** RMSD distributions during MD simulations of the DBD carrying the A19D (A), H35Y (B) and I86V (C) mutations and the DNA molecules in the corresponding complex models (D).


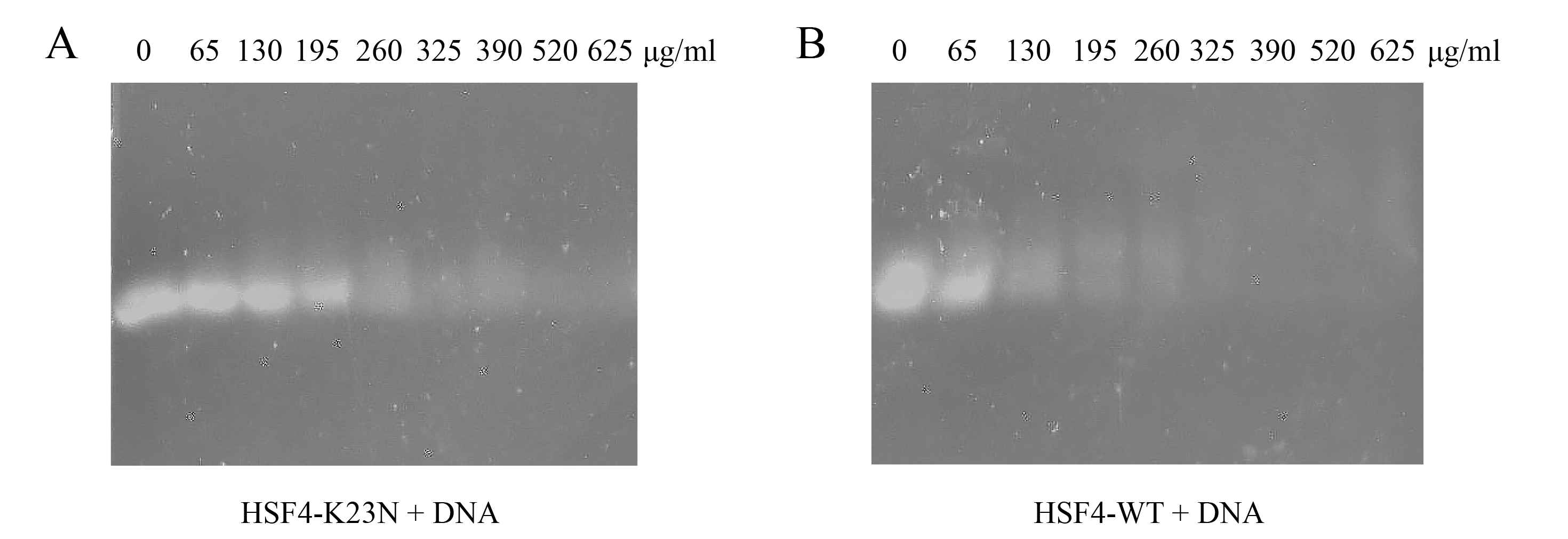


**Supplementary Figure 6.** The electrophoretic mobility shift assays (EMSAs) of DNA comprising three canonical HSE motifs incubated with the K23N mutant (**A**) and with wild-type HSF4 (**B**) at concentrations of 0, 65, 130, 195, 260, 325, 390, 520, 650 μg/ml from lane 1 to 9. The EMSA experiment was performed on 1% agarose gels, where the only band in each lane is the free DNA unbound by HSF4.
